# Identification of Microenvironment-Related Prognostic Genes in Bladder Cancer Based on Gene Expression Profile

**DOI:** 10.3389/fgene.2019.01187

**Published:** 2019-11-22

**Authors:** Yongxiang Luo, Guohua Zeng, Song Wu

**Affiliations:** ^1^Department of Urological Surgery, The Affiliated Luohu Hospital of Anhui University of Science and Technology, Shenzhen, China; ^2^Department of Urological Surgery, The First Affiliated Hospital of Guangzhou Medical University, Guangzhou, China; ^3^Shenzhen Following Precision Medical Institute, Shenzhen Luohu Hospital Group, Shenzhen, China

**Keywords:** bladder cancer, gene expression profile, microenvironment, immune scores, stromal scores

## Abstract

**Background and Objective:** Bladder cancer is the most common tumor in the urinary system, with a higher incidence in men than in women and a high recurrence rate. However, the mechanism of recurrence is still unclear, and it is urgent to clarify the pathophysiological mechanism of bladder cancer. To provide theoretical basis for the development of new therapies, investigating the effect of tumor microenvironment on the prognosis of bladder cancer is necessary.

**Methods:** We applied the Estimation of STromal and Immune cells in MAlignant Tumors using Expression data (ESTIMATE) algorithm to the downloaded TCGA (The Cancer Genome Atlas) transcriptome data to obtain the immune scores and stromal scores of each sample, and then divided the samples into two groups: high and low immune scores (or high and low stromal scores), and found that some differential genes were associated with poor prognosis of patients. We then performed protein-protein interaction (PPI) network analysis to explore the relationship between these differentially expressed genes. Moreover, we also performed (Gene Ontology) GO and (Kyoto Encyclopedia of Genes and Genomes) KEGG analyses to explore the potential functions of differentially expressed genes. Finally, our results were validated in an independent dataset.

**Results:** We identified 136 tumor microenvironment-related genes associated with poor prognosis of bladder cancer. GO annotation and KEGG pathway enrichment analysis found that these genes are mainly involved in extracellular matrix, Focal adhesion and phosphatidylinositol 3 kinase-protein kinaseB (PI3k-Akt) signaling pathway. Next, PPI network analysis revealed some hub genes including *Versican (VCAN), Thrombospondin 1 (THBS1) and Thrombospondin 1 (THBS2)*. Finally, 27 genes were further verified in the independent data set.

**Conclusions:** We found 27 tumor microenvironment-related genes of bladder cancer, which are associated with poor prognosis of bladder cancer. These genes may inspire researchers to develop new treatments for bladder cancer.

## Introduction

Bladder cancer (BLCA) is the most common cancer of the urinary system and is most common in men (Siegel et al., 2018). About 70–80% of bladder cancer cases are initially diagnosed as non-muscular invasive bladder cancer (NMIBC) (Alfred Witjes et al., 2017). For NMIBC patients, transurethral resection of the bladder tumor (TURBT) is the first choice. However, most of the postoperative NMIBC patients will relapse and progress to muscle invasive bladder cancer (MIBC), and the prognosis of MIBC patients is poor (Gao et al., 2018).

A growing body of evidence indicates that tumor microenvironment plays a very important role in tumor progression and metastasis (Hanahan and Weinberg 2011; Quail and Joyce 2013). The tumor microenvironment is composed of cancer cells and non-cancer cell components such as immune cells, stromal cells, endothelial cells, inflammatory cytokines, and extracellular matrix. Studies have shown that stromal cells in tumor microenvironment, such as fibroblasts, are related to the exocrine phenotype of T cells in bladder cancer (Mariathasan et al., 2018), while the presence of immune cells can mediate the killing of tumor cells through various mechanisms. In addition, non-immune cell components in tumor microenvironment also affect the treatment response. For example, transforming growth factor-β (TGF-β) secreted by fibroblasts can lead to the efflux of immune cells or the resistance of chemotherapy drugs (Wang et al., 2016). Therefore, the treatment effect of bladder cancer also changes with the degree of infiltration of stromal cells. Many studies have shown that tumor gene expression profile can quantify the immune activity in tumor microenvironment, such as the infiltration degree of CD8 (+) T cells (Sweis et al., 2016), so the gene expression profile of tumor tissue can reflect the relationship between tumor microenvironment and patient prognosis.

An algorithm ESTIMATE (Estimation of STromal and Immune cells in MAlignant Tumors using Expression data) has been applied to predict the infiltration level of stromal and immune cells by using single sample gene set enrichment analysis (ssGSEA) to calculate stromal and immune scores (Yoshihara et al., 2013). It has been proved that the ESTIMATE algorithm is effective for estimating stromal immune scores and tumor purity (Yoshihara et al., 2013; Zhang et al., 2017), thus providing a useful method for determining the prognosis of patients using gene expression profile of bladder cancer.

In this study, we downloaded 426 RNA sequencing datasets of bladder cancer in TCGA database, and then obtained stromal scores and immune scores of the bladder cancer datasets based on the ESTIMATE algorithm, and extracted a series of tumor microenvironment-related genes with poor prognosis. Finally, our results are verified in the Gene Expression Omnibus (GEO) data set.

## Materials and Methods

### Gene Expression Profile and Clinical Data

We first download Level3 gene expression profile for 426 cases of bladder cancer and clinical data from website (https://xenabrowser.net/datapages/), sequencing by Illumina HiSeq 2000 RNA Sequencing platform (**Supplementary Data Sheet S7**). Then, 165 cases of validation data set (GSE13507) downloaded from the GEO database (https://www.ncbi.nlm.nih.gov/geo/), which platform is GPL6102 Illumina human-6 v2.0 expression beadchip. Immune scores and stromal scores were obtained by the ESTIMATE algorithm using the estimate R package (Yoshihara et al., 2013) (R version 3.5.3).

### Analysis of Differentially Expressed Genes

We used the most commonly used R package limma to obtain differentially expressed genes (DEGs), which is the most effective and convenient method (Ritchie et al., 2015), where P value <0.01 and |logFC| > (mean(abs(logFC))+2*sd(abs(logFC)) were used as the cutoffs to filter the differentially expressed genes. R package pheatmap was performed to display the top 100 DEGs (https://cran.r-project.org/web/packages/pheatmap). 

### Survival Analysis

Survival analysis and univariable/multivariable Cox regression analysis were performed using the survival R package. The Kaplan-Meier survival curve was drawn to demonstrate the relationship between differential genes and overall survival, and the log-rank test was used to test the significance of the difference between the two. P < 0.05 was considered to be significant.

### Protein-Protein Interaction Network Analysis

Differential genes were imported into STRING database, which is an online database that searches for known protein interactions, to construct protein-protein interaction (PPI) network (https://string-db.org/). PPI network with node number greater than 10 was further analyzed by using MCODE plug-in in Cytoscape (version 3.7.1) software with default parameters (Bader and Hogue 2003).

### Gene Ontology and Kyoto Encyclopedia of Genes and Genomes Pathway Enrichment Analysis

In order to reveal the potential functions of differential genes, the R package clusterProfiler is used to perform Gene Ontology (GO) enrichment analysis and Kyoto Encyclopedia of Genes and Genomes (KEGG) pathway enrichment analysis, with p-value <0.05 as the cutoff value (Yu et al., 2012).

## Results

### Stromal Scores Was Significantly Correlated With Bladder Cancer Stage

**Figure 1:**
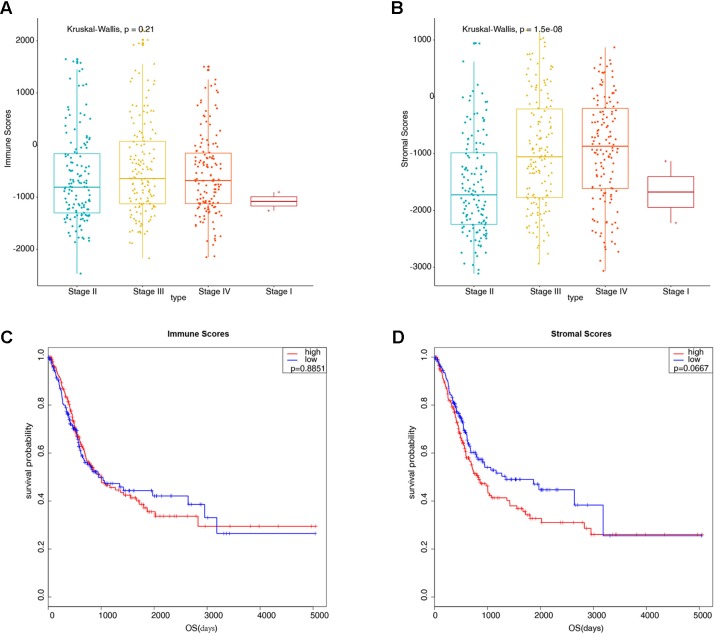
Preliminary exploration of the characteristics of stromal scores and immune scores in bladder cancer (BLCA). **(A)** Boxplots display the distribution of immune scores in bladder cancer pathological stages (P = 0.21). **(B)** Boxplots display the distribution of stromal scores in bladder cancer pathological stages (P = 1.5e-08). **(C)** Kaplan-Meier analysis of immune scores with bladder cancer patients (949 d *vs.* 1004 d, P = 0.8851). **(D)** Kaplan-Meier analysis of stromal scores with bladder cancer patients (1348 d *vs.* 823 d, P = 0.0667).

Gene expression profiles and clinical information data sets of 426 patients with bladder cancer were downloaded from the TCGA database. Some clinical information is shown in **Table 1**. A total of 424 cases of bladder cancer were obtained after not available (NA) value being removed for the following analysis. Based on the ESTIMATE algorithm, the stromal scores range from −3112.294 to 1170.948, and the immune scores range from −2467.750 to 2203.117 (**Figures 1A, B**). There was no significant difference in the mean immune scores of the four pathological stages (**Figure 1A**, P = 0.21), while there were significant differences in the stromal scores of the four stages, from low to high: Stage II and Stage I < Stage III < Stage IV (**Figure 1B**, P = 1.5e-08). Preliminary exploration showed that the stromal scores were correlated with the pathological stage of patients, the higher the stage of patients, the higher the stromal scores. To explore whether immune scores and stromal scores were correlated with survival time, 424 cases of bladder cancer were divided into two groups (low *vs.* high scores group) according to the scores, and 212 cases in each group were analyzed by Kaplan-Meier survival analysis. The results showed that the median survival time in the immune scores was no significant difference (**Figure 1C**, 949 d *vs.* 1004 d, P = 0.8851), and the median survival time in the low stromal scores group was higher than that in the high group (**Figure 1D**, 1348 d vs. 823 d, P = 0.0667), although there were no statistically significant.

**Table 1:** Clinical characteristics of TCGA data.

		TCGA data
Gender	Male	311 (73%)
	Female	115 (27%)
Stage	Stage I	2 (2NA) (0.1%)
	Stage II	134 (31.4%)
	Stage III	146 (34.2%)
	Stage IV	142 (33.3%)

### Differential Expressed Genes of Immune Scores and Stromal Scores

**Figure 2:**
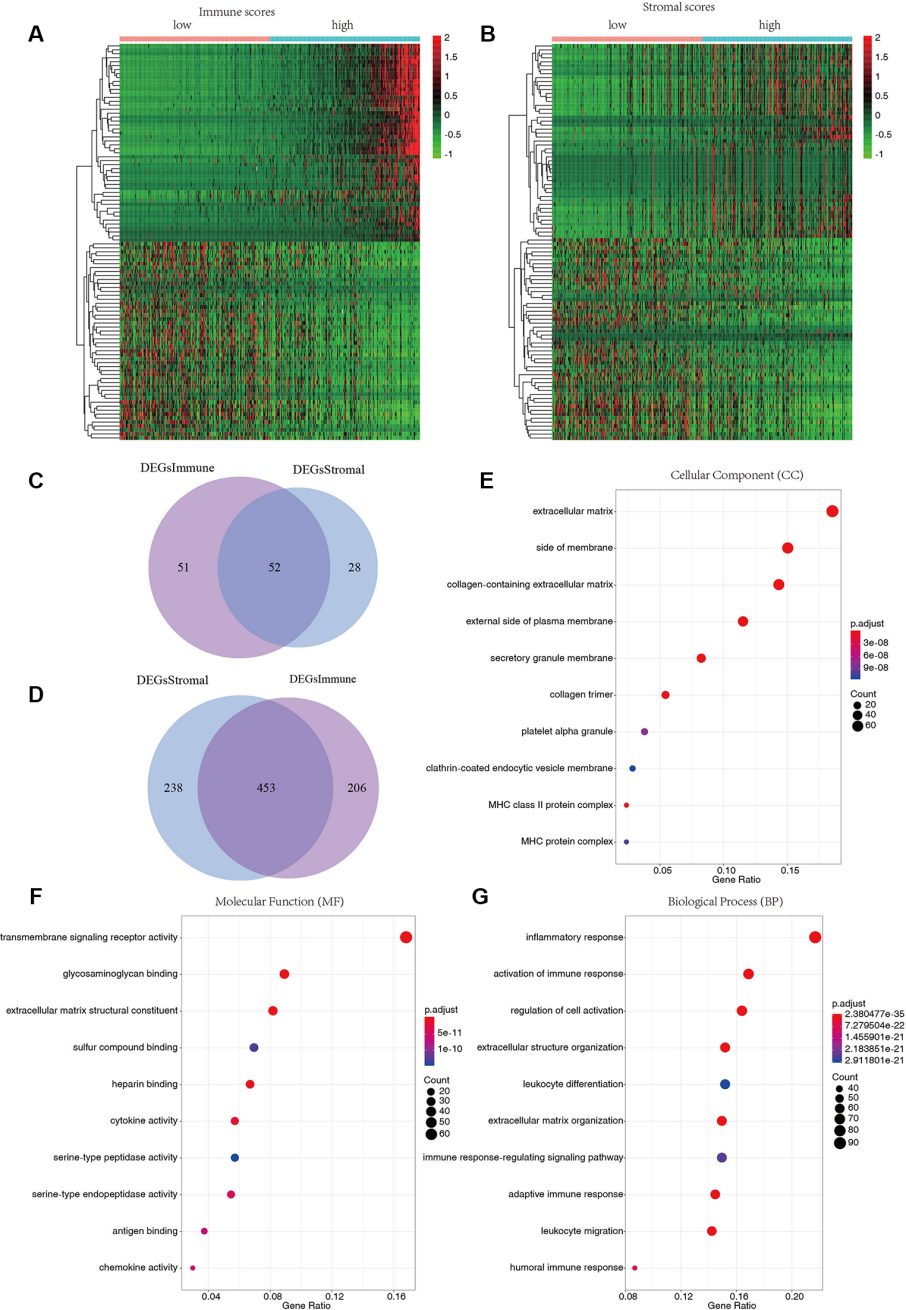
Differentially expressed genes of immune scores and stromal scores. **(A)** The heatmap of top 100 DEGs by comparing top half (high scores) with bottom half (low scores) of immune scores (P < 0.05, logFC > 1.41). **(B)** The heatmap of top 100 DEGs by comparing top half (high scores) with bottom half (low scores) of stromal scores (P < 0.05, logFC > 1.46). **(C**, **D)** Venn plots displaying co-upregulated and co-downregulated DEGs respectively. **(E**–**G)** Top 10 Gene Ontology (GO) terms (P < 0.05).

To reveal the relationship between transcriptome data and immune or stromal scores, gene expression profile from 424 TCGA patients were analyzed. Heatmaps show gene expression profiles of the top 100 DEGs obtained by differential expression analysis of low versus high immune scores or stromal scores group (**Figures 2A, B**). One hundred three up-regulated genes and 659 down-regulated genes were obtained based on the comparison of low and high immune scores (P < 0.01).Similarly, the low and high subsets of stromal scores were compared, and 80 up-regulated genes and 691 down-regulated genes were obtained (P < 0.01). The Venn plots showed 52 co-upregulation genes and 453 co-upregulation genes in the low immune scores and stromal scores groups (**Figures 2C, D**). Down-regulated genes were significantly more than the up-regulated genes among the differential genes obtained from the low and high grouping comparison. In addition, DEGs were generated by comparing low scores *vs.* high scores, downregulated genes were positive correlation with tumor stages. Therefore, 453 co-downregulated genes (**Supplementary Table S1**) with low stromal scores and low immune scores were selected for further analysis. Then 453 down-regulated genes were performed GO enrichment. The enriched GO terms found mainly include extracellular matrix, transmembrane signaling receptor activity, extracellular matrix structural beans, and extracellular structure organization (**Figures 2E–G**).

### Protein-Protein Interaction Network Analysis

**Figure 3:**
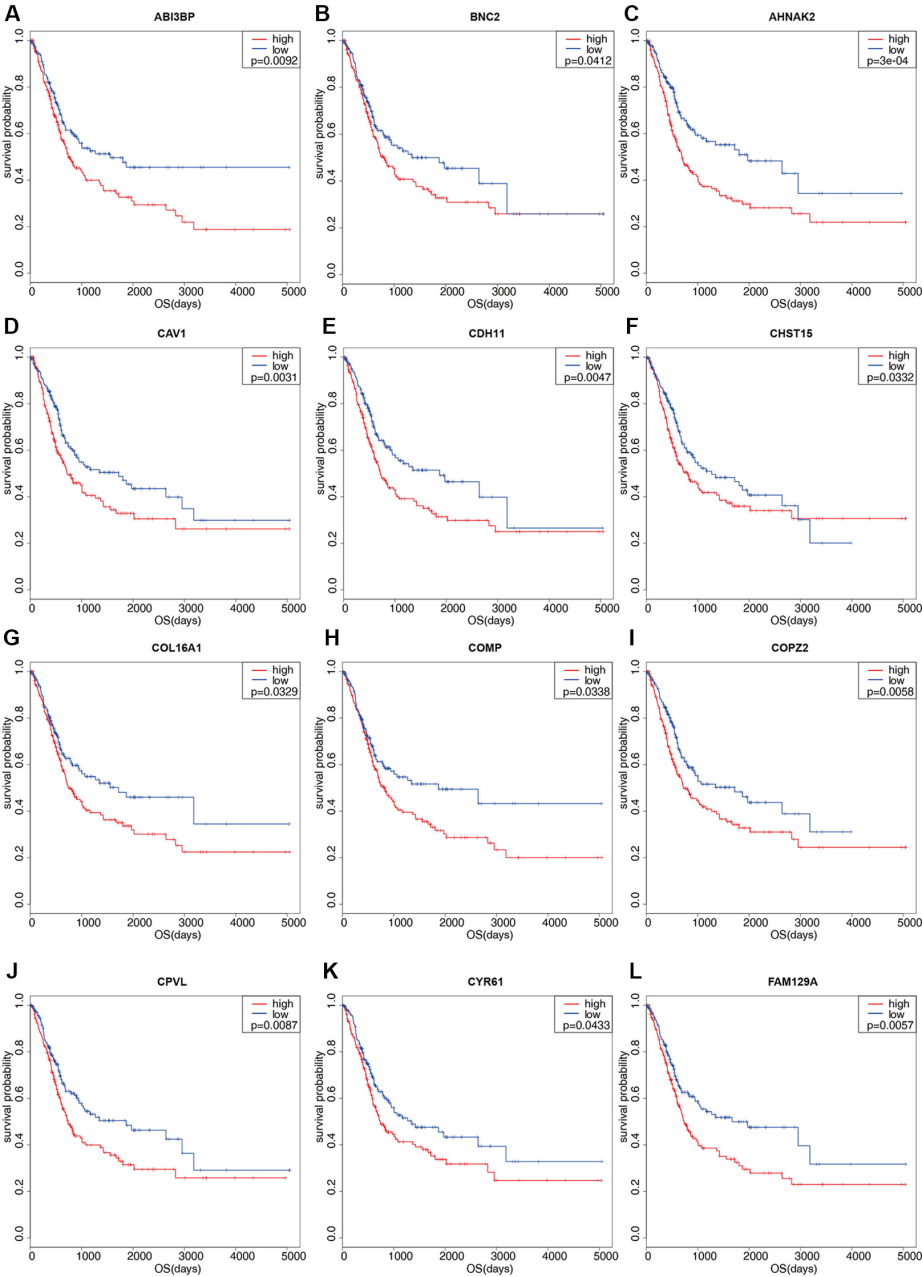
Kaplan-Meier survival plots of differential genes. Red line represents high stromal scores group and blue line represents low stromal scores group. P < 0.05 in log-rank test. OS (overall survival in days). **(A)** ABI3BP; **(B)** BNC2; **(C)** AHNAK2; **(D)** CAV1; **(E)** CDH11; **(F)** CHST15; **(G)** COL16A1; **(H)** COMP; **(I)** COPZ2; **(J)** CPVL; **(K)** CYR61; **(L)** FAM129A.

**Figure 4:**
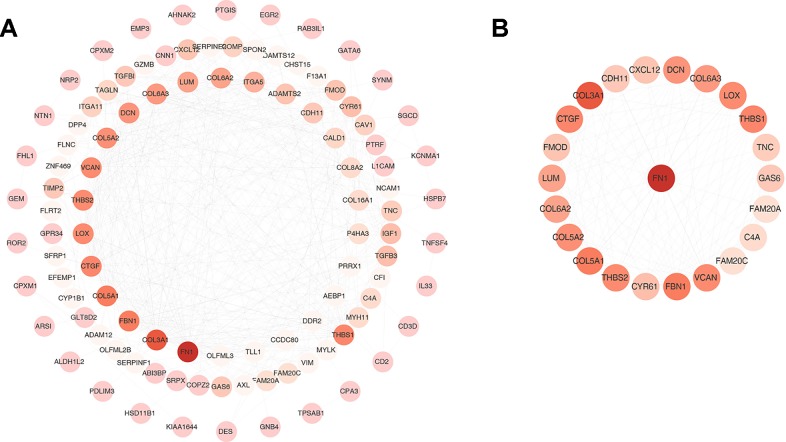
Protein-protein interaction (PPI) network and one clustering module. **(A)** The PPI network of 136 down-regulated differentially expressed genes (DEGs). **(B)** Module of the highest MCODE scores (MCODE score = 13.818). Node color deepens as the number of interacting proteins.

One hundred thirty six down-regulated DEGs related to survival time, which were generated by performing Kaplan-Meier survival analysis (selected genes are shown in **Figure 3**, **Supplementary Table S2**), were imported into STRING online database to build protein-protein interaction (PPI) network. The PPI network was composed of 103 nodes and 460 edges (**Figure 4A**). Important genes include *FN1*, *LOX*, *CTGF*, *FBN1*, *THBS1*, *THBS2*, and VCAN. Since these genes have the most connections with other genes, the more connections a gene has, the more important this gene is in the network. In addition, we conducted module analysis using MCODE to identify important modules in the PPI network and obtained a module with the highest score (Bader and Hogue 2003) (**Figure 4B**). The module network consists of 23 nodes and 152 edges. Consistently, important genes also include *FN1*, *THBS1*, *THBS2*, *FBN1*, *LOX*, *CTGF* and *VCAN*.

### Gene Ontology and Kyoto Encyclopedia of Genes and Genomes Pathway Enrichment Analysis

**Figure 5:**
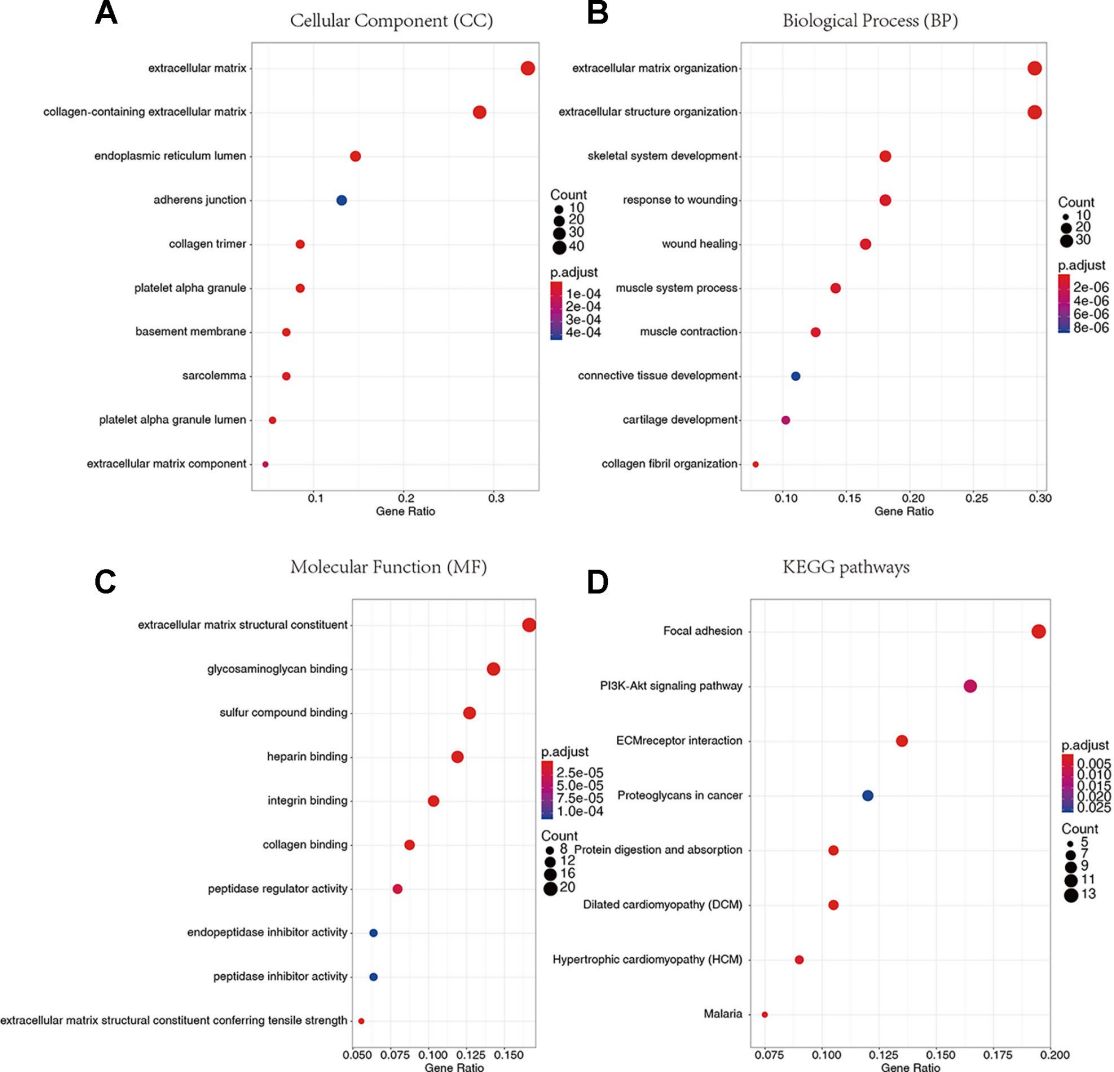
Gene Ontology (GO) terms and Kyoto Encyclopedia of Genes and Genomes (KEGG) pathway enrichment analysis. **(A)** Cellular Component. **(B)** Biological Process. **(C)** Molecular Function. **(D)** KEGG pathways.

We conducted GO and KEGG pathway enrichment analysis to explore the potential biological functions of 136 down-regulated genes. According to GO enrichment results, they are significantly enriched in the biological processes related to extracellular matrix. For example, extracellular matrix and collagen-containing extracellular matrix (**Figure 5A**), extracellular matrix organization and extracellular structural organization (**Figure 5B**), and extracellular matrix structure constituent (**Figure 5C**) and so on (**Supplementary Tables S3**–**S5**). In addition, in the enrichment analysis of KEGG pathways, they are mainly involved in regulating signaling pathways related to tumor progression, such as Focal adhesion, PI3K-Akt signaling pathway, extracellular matrix (ECM)-receptor and so on (**Figure 5D**, **Supplementary Table S6**).

### Validation in an Independent Data Set

To validate our results, GEO data containing 165 bladder cancer samples were used to assess the reproducibility of prognostic gene sets obtained from the TCGA database (Kim et al., 2010). A total of 27 genes associated with poor prognosis were verified (**Table 2**, **Figure 3**, **Supplementary Figure S1**). Subsequently, we performed univariable Cox analysis and multivariable Cox regression analysis on the 27 genes. As shown in the **Table 3**, these genes were divided into two groups (low-expression group *vs.* high-expression group), and the low expression group of these genes had a better prognosis than the high expression group in the univariable Cox analysis part (low-expression group *vs.* high-expression group, HR < 1, P < 0.05). However, multivariable Cox regression analysis showed that *AHNAK2* gene was significantly associated with overall survival after analysis using the combination of these genes (low-expression group *vs.* high-expression group, HR = 0.64, P = 0.037).

**Table 2:** Validated genes in GEO dataset.

Categories	Gene symbols
Cell adhesion	*CDH11, COL16A1, COMP, TGFBI, THBS1, THBS2, VCAN*
Extracellular matrix	*ABI3BP, SERPINE2, SERPINF1, TGFB3*
Plasma membrane	*FAM129A, PTGIS, SYNM, TNFAIP8L3*
Carboxypeptidase Tissue development Cytoplasm	*CPVL BNC2, CAV1, MYADM, MYH11, SULF2AHNAK2, CHST15, COPZ2, MSRB3, CYR61, GYPC*

**Table 3:** Univariable and multivariable Cox regression analysis of the 27 genes associated with poor prognosis.

Variables (low *vs.* high)	Univariable analysis			Multivariable analysis		
	HR	95% CI of HR	P	HR	95% CI of HR	P
ABI3BP	0.68	0.51–0.91	0.01	0.93	0.61–1.42	0.729
AHNAK2	0.58	0.43–0.78	<0.001	0.64	0.41–0.97	0.037
BNC2	0.74	0.55–0.99	0.042	1.87	1.02–3.43	0.043
CAV1	0.65	0.49–0.87	0.003	0.79	0.53–1.19	0.262
CDH11	0.66	0.49–0.88	0.005	0.9	0.45–1.79	0.769
CHST15	0.73	0.55–0.98	0.034	1.12	0.72–1.73	0.627
COL16A1	0.73	0.54–0.98	0.034	1.21	0.76-–1.92	0.42
COMP	0.73	0.54–0.98	0.035	0.94	0.61–1.46	0.796
COPZ2	0.67	0.5–0.89	0.006	0.99	0.62–1.59	0.974
CPVL	0.68	0.51–0.91	0.009	0.9	0.6–1.34	0.599
CYR61	0.74	0.56–0.99	0.044	1.06	0.7–1.62	0.777
FAM129A	0.66	0.5–0.89	0.006	0.91	0.61–1.35	0.625
GYPC	0.67	0.5–0.9	0.007	0.88	0.51–1.51	0.642
MSRB3	0.62	0.46–0.83	0.001	0.63	0.36–1.11	0.112
MYADM	0.69	0.51–0.92	0.011	1.06	0.66–1.7	0.809
MYH11	0.74	0.55–0.99	0.042	1.29	0.8–2.07	0.292
PTGIS	0.66	0.5–0.89	0.006	0.87	0.55–1.39	0.573
SERPINE2	0.61	0.46–0.82	0.001	0.7	0.49–1.01	0.055
SERPINF1	0.68	0.51–0.91	0.01	1	0.59–1.68	0.987
SULF2	0.74	0.55–0.99	0.041	1.23	0.77–1.97	0.396
SYNM	0.69	0.51–0.92	0.012	1.02	0.65–1.58	0.935
TGFB3	0.66	0.49–0.88	0.005	0.94	0.56–1.57	0.816
TGFBI	0.71	0.53–0.95	0.022	1.19	0.77–1.84	0.441
THBS1	0.73	0.55–0.98	0.034	1.14	0.75–1.74	0.537
THBS2	0.68	0.5–0.91	0.009	0.67	0.42–1.06	0.087
TNFAIP8L3	0.62	0.46–0.83	0.001	0.82	0.51–1.3	0.389
VCAN	0.74	0.55–1	0.049	1.08	0.65–1.79	0.771

## Discussion

**Figure 6:**
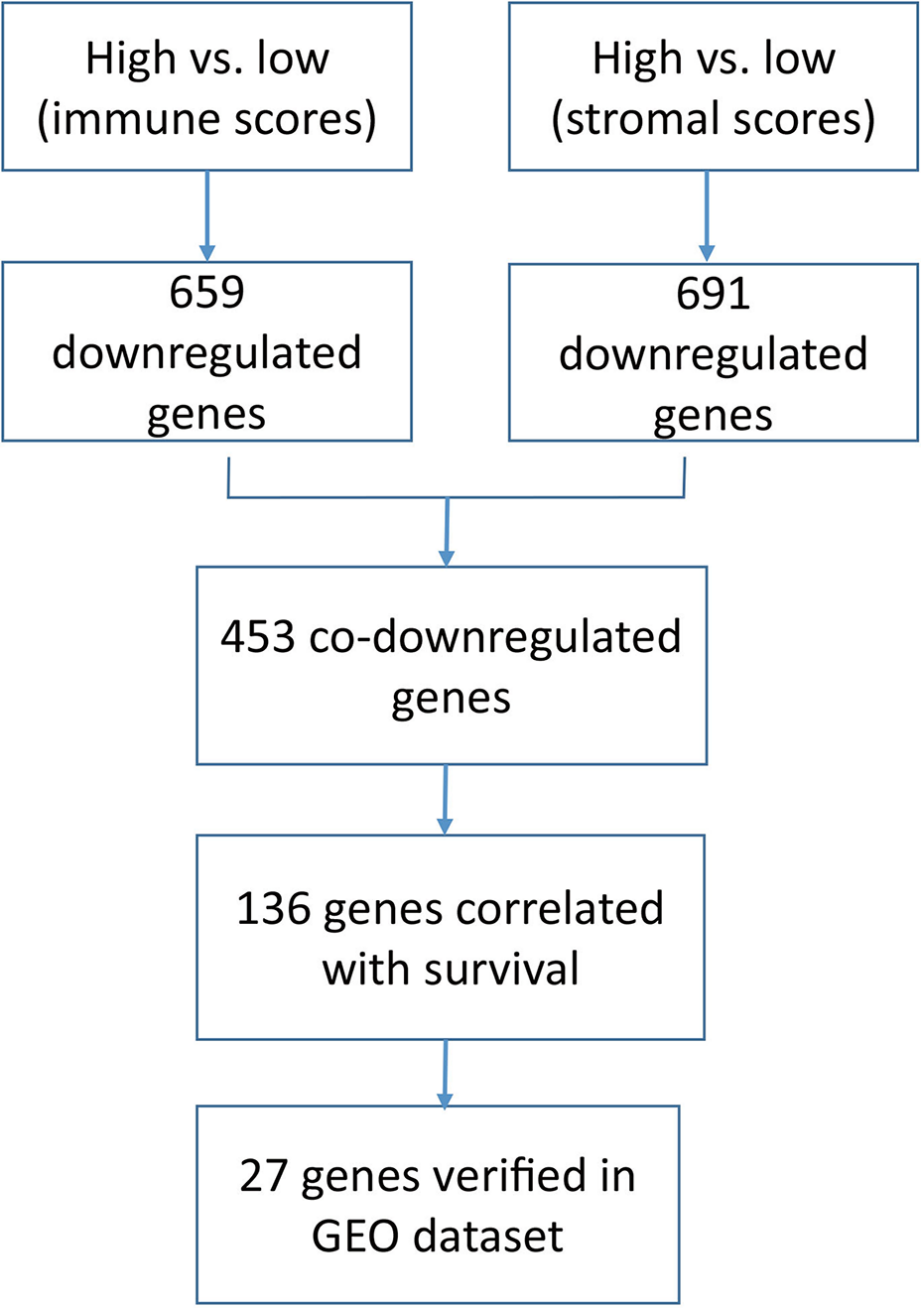
Workflow of our current research.

In this study, we identified 136 tumor microenvironment-related genes that contribute to the overall survival time of BLCA. In the end, 27 genes were identified in an independent GEO dataset (workflow, **Figure 6**).

First, we found that the stromal scores gradually increased from Stage II to Stage IV, indicating that the stromal components in the tumor microenvironment may play an important role in the process of tumor progression. Consistent with previous studies, tumor stromal component contributes to tumor budding, epithelial mesenchymal transformation and lymph node metastasis (De Wever and Mareel 2003; Li et al., 2016). Secondly, we analyzed 453 differentially expressed genes obtained from comparison of high *vs.* low immune scores (or stromal scores) groups, and found that most of them were tumor microenvironment-related genes (**Figure 2**). Next, survival analysis of 453 differentially expressed genes identified 136 genes associated with poor prognosis of BLCA. Finally, 27 tumor microenvironment-related genes significantly correlated with prognosis were obtained by cross-validation of GEO data set in 165 cases of bladder cancer (**Table 2**).

Among the 27 genes, *VCAN*, *THBS1* and *THBS2* had more connections in PPI (**Figure 4**). *VCAN* belongs to the proteoglycan family and encodes chondroitin sulfate proteoglycan, which is a highly conserved structural component in ECM and is expressed in invasive and metastatic cancers (Wu et al., 2008; Kischel et al., 2010). Said et al. found that high expression of *VCAN* predicted poor prognosis in patients with bladder cancer (Said et al., 2012). In addition, *VCAN* has four subtypes, V0, V1, V2, and V3, and all of which contribute to the proliferation, adhesion, and migration of tumor cells and regulate their interaction with tumor microenvironment (Chiodoni et al., 2010). *THBS1* and *THBS2* belong to the glycoprotein family. Some studies have reported that *THBS1* can stimulate the malignant invasiveness of cancer (Firlej et al., 2011; Horiguchi et al., 2013; Pal et al., 2016). About *THBS-2*, it has been reported that its expression is positively correlated with malignant invasiveness and poor prognosis in BLCA patients (Chang et al., 2016). In conclusion, we used bioinformatics methods to identify a series of genes related to tumor microenvironment, and these genes are related to patients’ prognosis. Although these genes were verified in the GEO independent dataset, further analysis and validation of integrating multiple omics data such as mutation, copy number variation, and DNA methylation data should be needed. In addition, since our study is based on data analysis, biological experiments are needed to further verify our results.

## Conclusion

We found some tumor microenvironment-related genes through multiple analyses, which may lead to poor clinical outcomes in patients. Our study may provide new ideas for the development of new therapies for stromal component in bladder cancer.

## Data Availability Statement

Publicly available datasets were analyzed in this study. This data can be found here: https://tcga.xenahubs.net/download/TCGA.BLCA.sampleMap/HiSeqV2.gz, https://tcga.xenahubs.net/download/TCGA.BLCA.sampleMap/BLCA_clinicalMatrix.gz, ftp://ftp.ncbi.nlm.nih.gov/geo/series/GSE13nnn/GSE13507/soft/.

## Author Contributions

YL: conception, design, and performance of the research and writing of the paper. SW: supervision of the research. GZ: provision of suggestions in figure preparation. All authors read and approved the final version of the manuscript.

## Funding

The study was financially supported by Shenzhen Basic Research Project (JCYJ20160429172247015, JCYJ20160429093033251), The National Natural Science Foundation Fund of China (81672533) and Special Funds for Strategic Emerging Industries Development in Shenzhen (20180309163446298).

## Conflict of Interest

The authors declare that the research was conducted in the absence of any commercial or financial relationships that could be construed as a potential conflict of interest.
