# Supplementary figures and images for: Identification of Microenvironment-Related Prognostic Genes in Bladder Cancer Based on Gene Expression Profile
Source: Front Genet. 2019 Nov 22;10:1187. doi: 10.3389/fgene.2019.01187 (PMC6883806; doi:10.3389/fgene.2019.01187)

GYPC

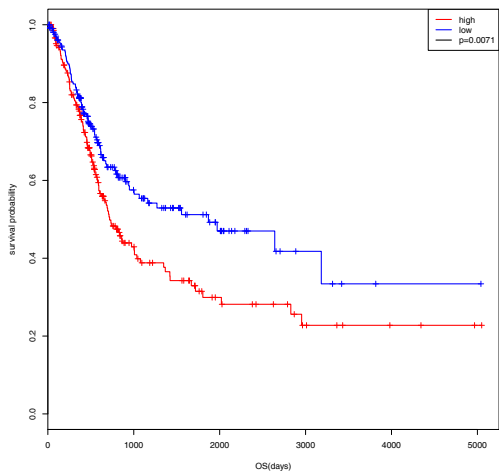

MSRB3

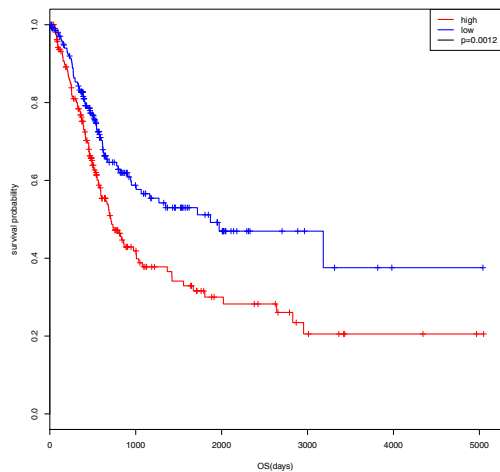

MYADM

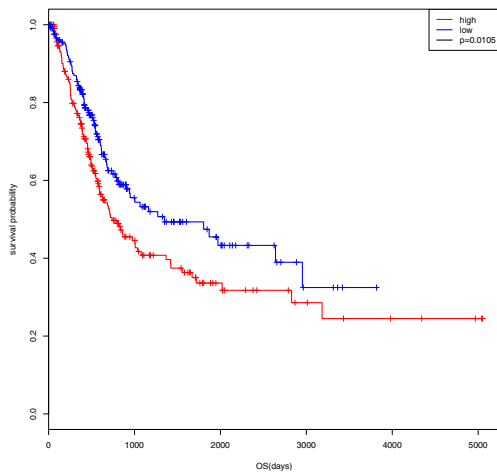

MYH11

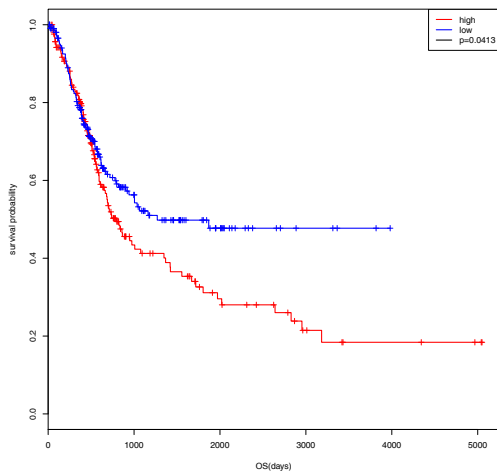

PTGIS

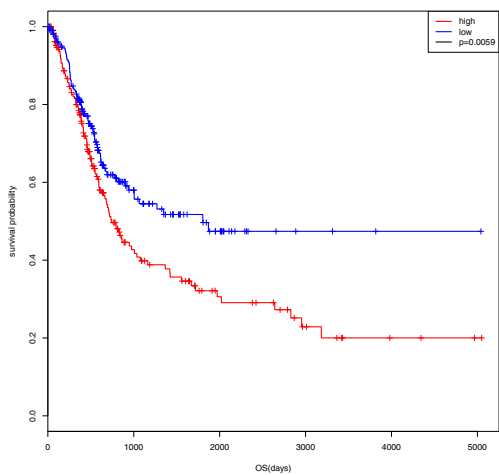

SERPINE2

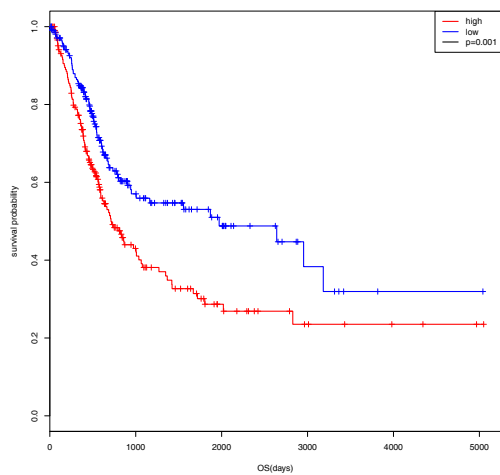

SERPINF1

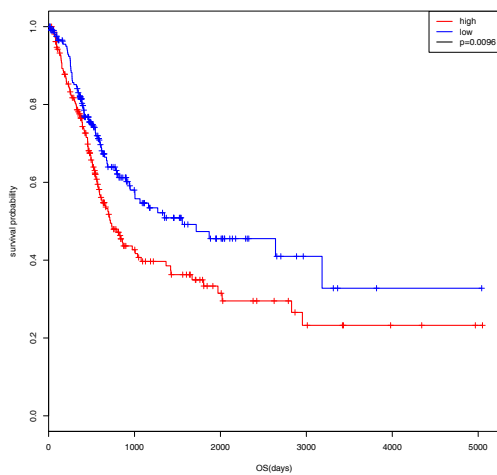

SULF2

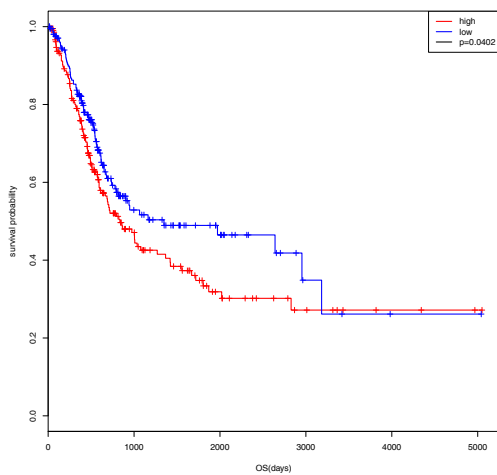

SYNM

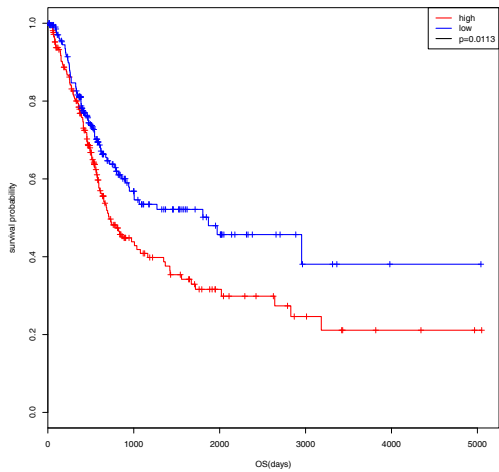

TGFB3

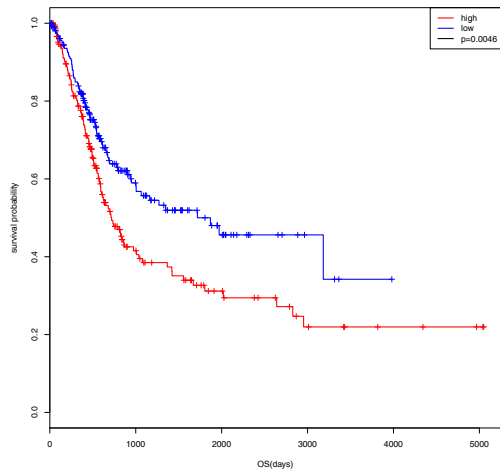

TGFB1

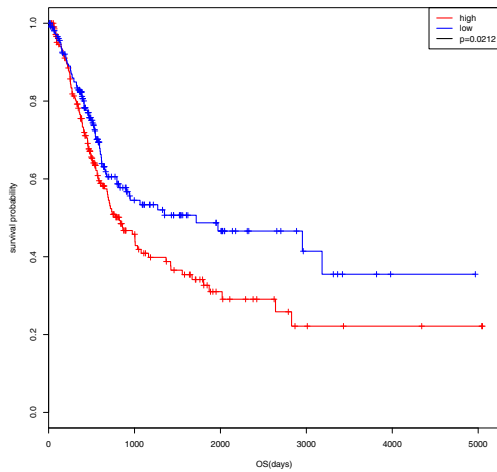

THBS1

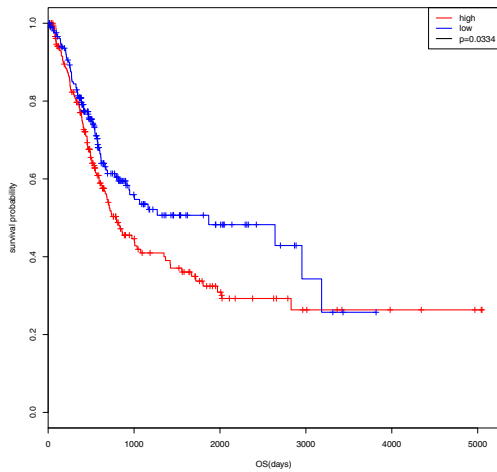

THBS2

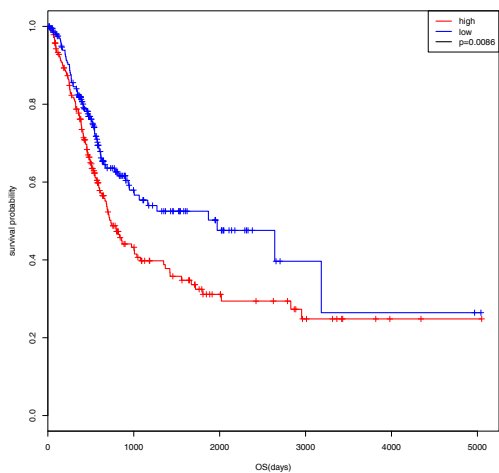

TNFAIP8L3

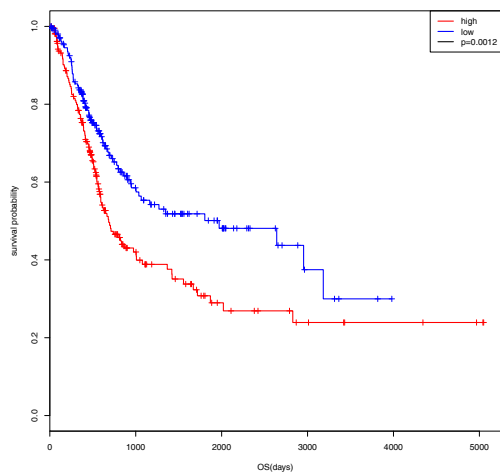

VCAN

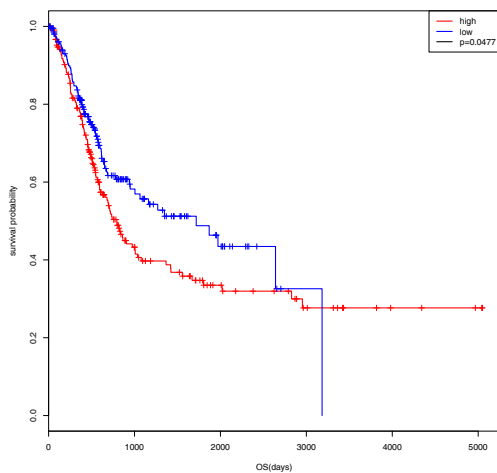

Supplement: Supplementary file 8 [file Image_1.pdf]
