# Supplementary material for: Identification of Microenvironment-Related Prognostic Genes in Bladder Cancer Based on Gene Expression Profile
Source: Front Genet. 2019 Nov 22;10:1187. doi: 10.3389/fgene.2019.01187 (PMC6883806; doi:10.3389/fgene.2019.01187)
Supplement: Supplementary file 9 [file Table_1.docx]

# Supplementary Data

**Supplementary Data Sheet S1.** 453 co-downregulated genes.

**Supplementary Data Sheet S2.** 136 down-regulated differential genes correlated with overall survival days.

**Supplementary Data Sheet S3.** Enriched biological process GO terms.

**Supplementary Data Sheet S4.** Enriched cellular component GO terms.

**Supplementary Data Sheet S5.** Enriched molecular function GO terms.

**Supplementary Data Sheet S6.** KEGG pathway enriched terms.

**Supplementary Data Sheet S7 - Raw Data and Code.** GEO and TCGA RNA sequencing level3 data.

# Supplementary Figures and Tables

**Supplementary Data Sheet S8 -** Figure S1. Kaplan-Meier survival plots of 15 validated genes in GEO datasets
